# Description of a New Species of the *Pareas hamptoni* Complex from Yunnan, China, with Confirmation of *P. hamptoni* Sensu Stricto in China (Squamata, Pareidae) †

**DOI:** 10.3390/ani14030421

**Published:** 2024-01-27

**Authors:** Shuo Liu, Mingzhong Mo, Mei Li, Biao Li, Xiong Luo, Dingqi Rao, Song Li

**Affiliations:** 1 Kunming Natural History Museum of Zoology, Kunming Institute of Zoology, Chinese Academy of Sciences, Kunming 650223, China; 2 Honghe Prefecture Forestry and Grassland Bureau of Yunnan Province, Mengzi 661199, China; 3 Guanyinshan Provincial Nature Reserve Management and Protection Bureau, Yuanyang 662400, China; 4 Kunming Institute of Zoology, Chinese Academy of Sciences, Kunming 650223, China; 5 Yunnan Key Laboratory of Biodiversity Information, Kunming Institute of Zoology, Chinese Academy of Sciences, Kunming 650201, China

**Keywords:** Cyt b, morphology, ND4, *Pareas guanyinshanensis* sp. nov., taxonomy

## Abstract

**Simple Summary:**

In this paper, a new *Pareas* species of the *P. hamptoni* complex is described from southern Yunnan Province, China, based on morphological and molecular evidence. Genetically, the new species is most closely related to *P. hamptoni* sensu stricto, for which we confirm the distribution in China. Morphologically, the new species can be distinguished from *P. hamptoni* sensu stricto and all other congeners by a combination of morphological characteristics. The discovery of the new species brings the total number of recognized species of the genus *Pareas* to 31, of which 25 occur in China.

**Abstract:**

We describe a new species of the genus *Pareas*, based on three specimens collected from Guanyinshan Provincial Nature Reserve in Yuanyang County, Honghe Prefecture, Yunnan Province, China. The new species is distinguished from its congeners by one preocular, one postocular or postocular fused with subocular, loreal not bordering the orbit, one row enlarged vertebral scales, five rows keeled mid-dorsal scales at the middle of the body, 189–192 ventral scales and 72–89 subcaudal scales. The dorsal surfaces of the head and body are yellowish red or yellowish brown, and the belly and ventral surfaces of the head and tail are pinkish yellow or yellow with more or less small black spots. Phylogenetic analyses of mitochondrial DNA recovered the new species being the sister taxon to *P. hamptoni* sensu stricto. The genetic divergences between the new species and *P. hamptoni* sensu stricto were 4.2% in the Cyt b sequences and 5.0% in the ND4 sequences. In addition, based on specimens collected from Honghe and Wenshan prefectures, we confirmed that *P. hamptoni* sensu stricto is distributed in China.

## 1. Introduction

The family Pareidae Romer, 1956 is an ancient lineage, but there is as yet practically no fossil record known for this group [1]. Zheng and Wiens [2] and Deepak et al. [3] estimated that Pareidae split from other caenophidians during the Paleocene, while Zaher et al. [4] inferred that this happened during the Eocene. Deepak et al. [3] and Poyarkov et al. [5] estimated that Pareidae divided into two subfamilies, namely, Pareatinae Romer, 1956 and Xylophiinae Deepak, Ruane and Gower, 2019, during the middle Eocene. Xylophiinae encompasses one genus, namely, *Xylophis* Beddome, 1878, while Pareatinae encompasses three genera, namely, *Aplopeltura* Duméril, 1853, *Asthenodipsas* Peters, 1864, and *Pareas* Wagler, 1830 [3,5,6].

The genus *Pareas* split from its sister genus *Aplopeltura* during the Oligocene [3,5]. *Pareas* covers a group of highly specialized snakes that only feed on snails or slugs [7,8,9,10,11]. This special habit also gives them a significant advantage in the niche, so that they occupied a vast area from Sundaland to northeastern India and southern China and evolved into a large number of different species [5,6,10,12,13,14,15,16].

Hampton’s slug-eating snake, *Pareas hamptoni* (Boulenger, 1905), was originally described based on a single specimen from Mogok, Myanmar [17]. Subsequently, this species was thought to be widely distributed, ranging across mainland Southeast Asia and southern China [18,19,20,21]. Thereafter, Wang et al. [6] demonstrated that the populations previously identified as *P. hamptoni* in southeast China belong to *P. formosensis* (Van Denburgh, 1909). Ding et al. [11] described the population that had previously been identified as *P. hamptoni* in south central Yunnan, China as a new species, *P. geminatus* Ding, Chen, Suwannapoom, Nguyen, Poyarkov & Vogel, 2020. They demonstrated that the populations previously identified as *P. hamptoni* in northeast, central, and central south Vietnam belong to *P. formosensis*, whereas the populations in northwest Vietnam belong to *P. hamptoni* sensu stricto. Liu and Rao [12] described the population that had previously been considered *P. hamptoni* from southwestern Yunnan, China, as a new species, *P. xuelinensis* Liu & Rao, 2021. Until now, whether *P. hamptoni* sensu stricto is distributed in China has been a matter of uncertainty.

During our field research in southern Yunnan Province, China, from 2019 to 2023, we collected a series of *Pareas* specimens that closely resemble *P. hamptoni*. However, further comprehensive analyses of their molecular and morphological characteristics have demonstrated that these specimens do not belong to the same species but are from two separate taxa. One corresponds to *P. hamptoni* sensu stricto, while the other is different from all known congeners. Therefore, we herein confirm the distribution of *P. hamptoni* sensu stricto in China and describe the unique taxon as a new species.

## 2. Materials and Methods

### 2.1. Sampling

Specimens were caught by hand at night. Photographs were taken in situ before the specimens were collected. Liver tissues were cut and stored in analytical pure ethanol and the snakes were preserved in a 75% concentration of ethanol. Specimens were deposited at Kunming Natural History Museum of Zoology, Kunming Institute of Zoology, Chinese Academy of Sciences (KIZ).

### 2.2. Morphometrics

Measurements were taken to the nearest 1 mm with a ruler. Paired meristic characteristics are given as left/right. The methodology of measurements and meristic counts is the same as that used by Liu et al. [22]. The abbreviations are as follows: anterior temporals (ATem); dorsal scale rows (DS), counted at one head length behind the head-mid-body-one head length before the vent; infralabials (InfL); loreal bordering orbit (LoBO); maxillary teeth (Max); the number of enlarged dorsal scale rows at mid-body (NED); the number of keeled dorsal scale rows (NKD), counted at one head length behind the head-mid-body one head length before the vent; postoculars (PosO); precloacal plate (Prec); preoculars (PreO); prefrontal bordering orbit (PrFBO); posterior temporals (PTem); subcaudals (Sc); subocular-postocular fused (SPOF); suboculars (SubO); supralabials (SupL); snout–vent length (SVL); tail length (TL); and ventrals (Vs).

### 2.3. Molecular Analyses

Molecular data were generated for nine specimens collected from Honghe and Wenshan prefectures, Yunnan Province, China. Other sequences used in the phylogenetic analyses were obtained from GenBank. All new sequences have been deposited in GenBank. *Aplopeltura boa* (Boie, 1828) was selected as the outgroup according to Liu et al. [23]. Details of all the sequences used in this study can be found in Table 1.

Total genomic DNA was extracted from liver tissue samples. The sequences of the mitochondrial genes, cytochrome b (Cyt b) and NADH dehydrogenase subunit 4 (ND4), were amplified by a polymerase chain reaction (PCR) using the primers L14910/H16064 [24] and ND4F/ND4LEUR [25], respectively. The amplification products were purified and sequenced by Tsingke Biotechnology Co., Ltd. (Beijing, China). The sequences were stitched using SeqMan in Lasergene 7.1 [26].

The sequences were aligned using ClustalW [27] that is integrated in MEGA 11 [28] with the default parameters. The genetic divergences (uncorrected p-distance) between species were calculated using MEGA 11 [28]. The best substitution models were selected with ModelFinder [29], using the Bayesian information criterion (BIC). Phylogenetic analyses were constructed based on the concatenated sequences of the Cyt b and ND4 gene sequences. Bayesian inference was performed with MrBayes 3.2.6 [30], using the GTR+F+I+G4 model for the first and second codon positions of Cyt b and ND4 and the GTR+F+G4 model for the third codon positions of Cyt b and ND4. Maximum likelihood analysis was performed with IQ-TREE 1.6.12 [31], using the GTR+F+I+G4 model for the first and second codon positions of Cyt b and ND4 and the TN+F+R3 model for the third codon positions of Cyt b and ND4.

## 3. Results

### 3.1. Morphological Results

Meristic and mensural characteristics were noted for all newly collected specimens (Table 2 and Table 3). The morphological characteristics of the specimens collected from Malipo County in Wenshan Prefecture and Hekou and Jianshui counties in Honghe Prefecture agree with the morphological characteristics of *Pareas hamptoni* sensu stricto given by Ding et al. [11]. However, the morphological characteristics of the specimens collected from Yuanyang County, Honghe Prefecture, can be distinguished from those of the specimens collected from Malipo, Hekou, and Jianshui, and all other named species of *Pareas*.

### 3.2. Molecular Results

**Figure 1:**
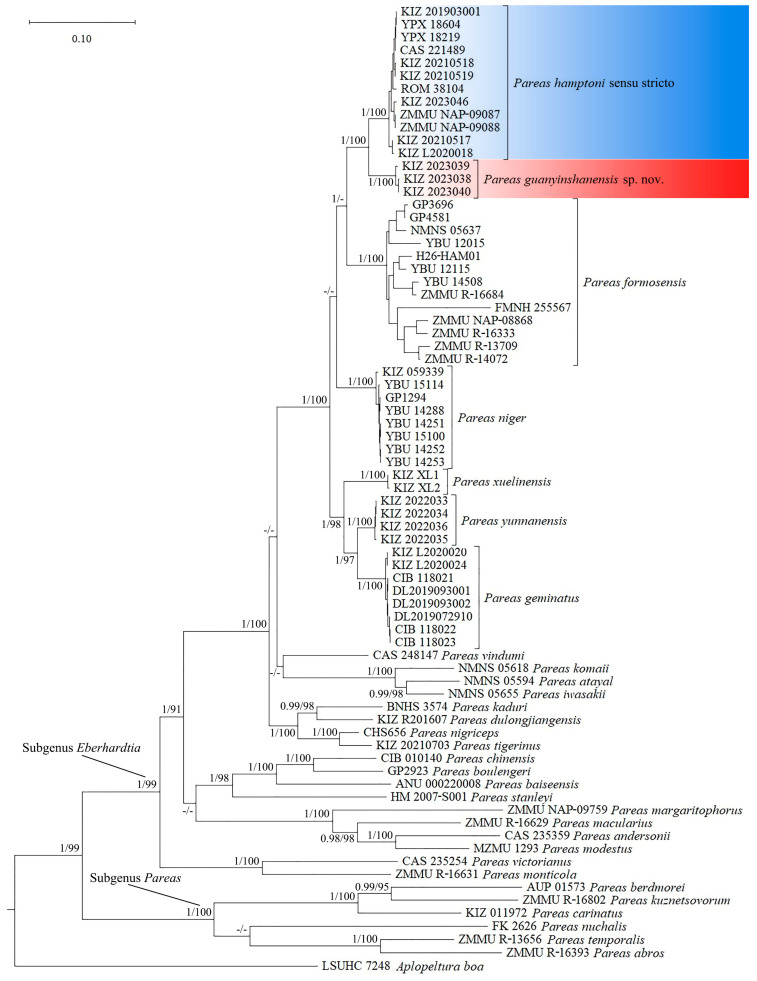
Bayesian phylogeny tree of *Pareas*, based on concatenated Cyt b and ND4 fragments. Node numbers before “/” indicate Bayesian posterior probabilities (values below 0.90 are not shown) and numbers after “/” indicate ultrafast bootstrap support for the maximum likelihood analyses (values below 90 are not shown).

Bayesian inference and maximum likelihood analysis resulted in similar topologies; the specimens from Malipo, Hekou, and Jianshui were clustered with *Pareas hamptoni* sensu stricto from Myanmar and Vietnam with strong supports, which should also be assigned to *P. hamptoni* sensu stricto, while the specimens from Yuanyang formed a distinct lineage sister to *P. hamptoni* sensu stricto with strong supports (Figure 1). The genetic divergences (uncorrected p-distance) between the specimens from Yuanyang and *P. hamptoni* sensu stricto were 4.2% and 5.0% in the Cyt b and ND4 gene sequences, respectively (Table 4 and Table 5).

### 3.3. Systematics

Class Reptilia Laurenti, 1768

Order Squamata Oppel, 1811

Suborder Serpentes Linnaeus, 1758

Infraorder Caenophidia Hoffstetter, 1939

Family Pareidae Romer, 1956

Subfamily Pareinae Romer, 1956

Genus *Pareas* Wagler, 1830

Subgenus *Eberhardtia* Angel, 1920

*Pareas guanyinshanensis* sp. nov.

urn:lsid:zoobank.org:act:AC079EB5-1E12-4AB6-BB55-666CD66D3C0F

**Figure 2:**
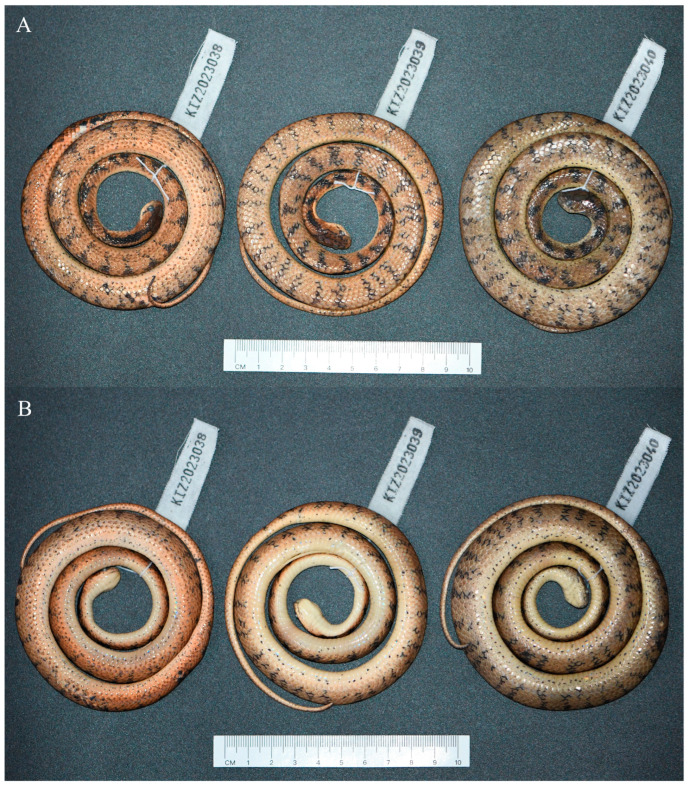
The type series of *Pareas guanyinshanensis* sp. nov. in preservative. (**A**) Dorsal view; (**B**) ventral view.

**Figure 3:**
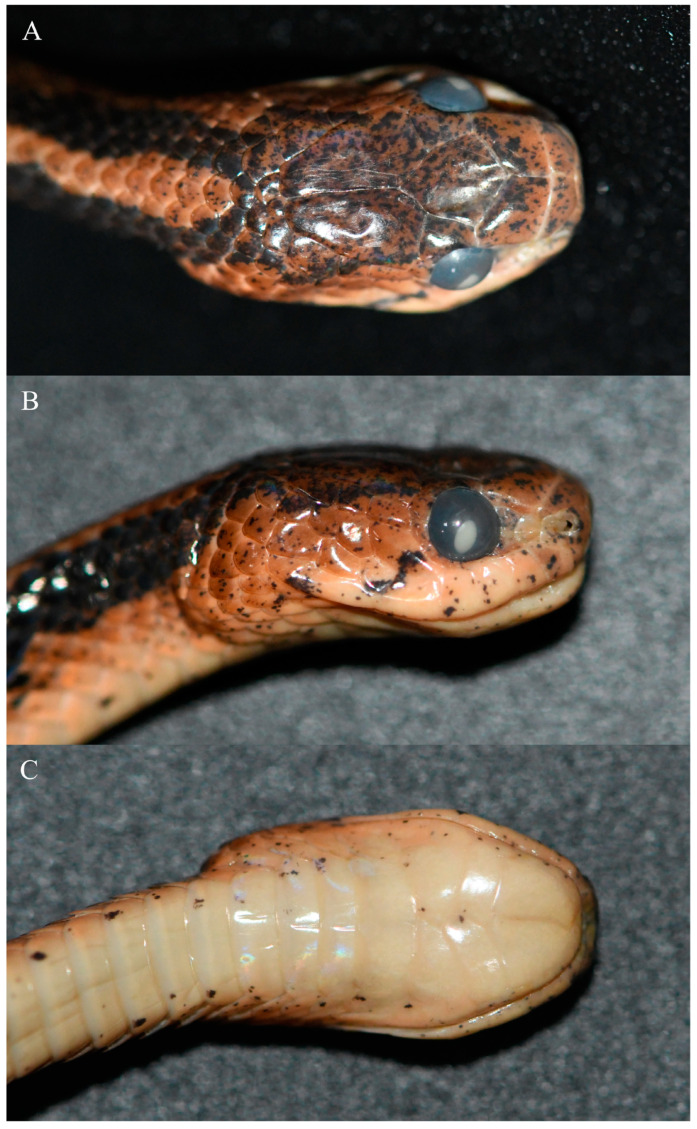
Close-up views of the head of the holotype (KIZ 2023038) in preservative. (**A**) Dorsal view; (**B**) right side view; (**C**) ventral view.

**Figure 4:**
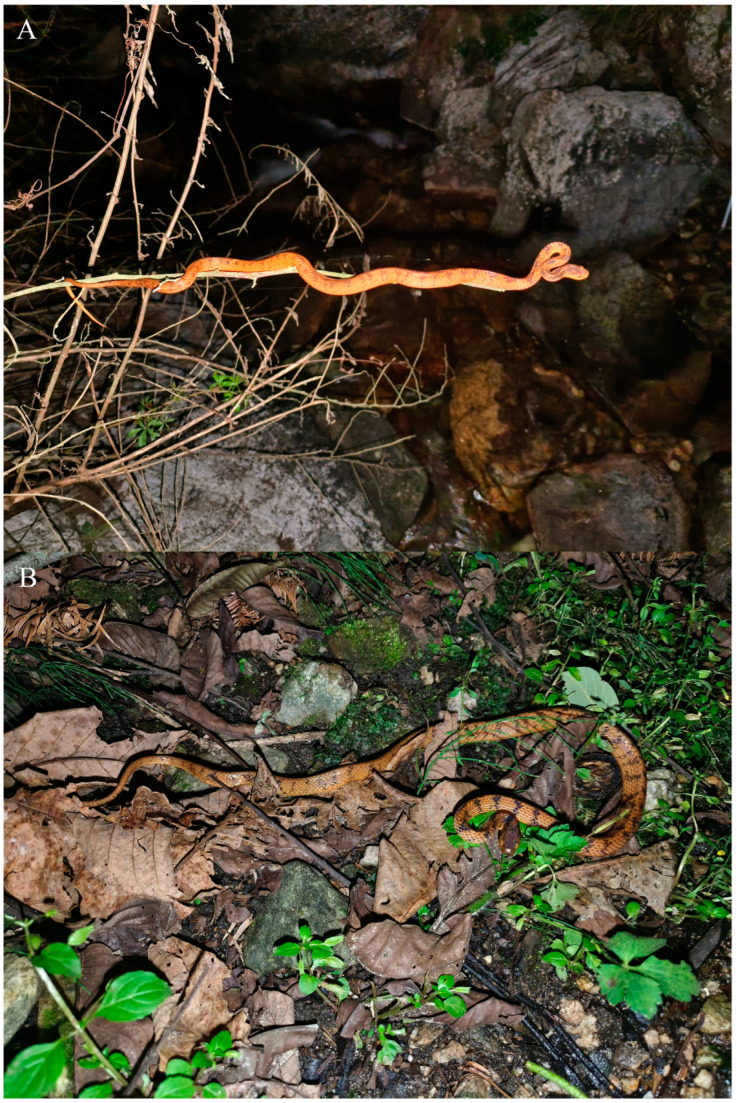
*Pareas guanyinshanensis* sp. nov. in life. (**A**) The holotype (KIZ 2023038); (**B**) the paratype (KIZ 2023040).

Figure 2, Figure 3 and Figure 4

Holotype. KIZ 2023038, adult female, collected from Guanyinshan Provincial Nature Reserve, Shuijingwan Village, Ganiang Township, Yuanyang County, Honghe Prefecture, Yunnan Province, China (23°3′5″ N, 102°51′54″ E; 1750 m a.s.l.) on 17 May 2023 by Shuo Liu.

Paratypes. KIZ 2023039–2023040, two adult females, with the same collection data as the holotype.

Diagnosis. SVL 482–540 mm, TL/SVL 0.26–0.30; prefrontal bordering orbit; loreal not bordering orbit; 1 preocular; 1 postocular or postocular fused with subocular; 7–8 supralabials; 6–8 infralabials; infralabial not fused with chin-shield; 3 chin-shield pairs; dorsal scales in 15 rows throughout; 1 row of vertebral scales enlarged; scales not keeled at anterior part of body, 5 rows of mid-dorsal scales keeled at middle part of body, 5–7 rows of mid-dorsal scales keeled at posterior part of body; ventral scales 189–192; subcaudals 72–89, paired; precloacal plate undivided; maxillary teeth 4–5. Dorsal surface of head dark yellowish red or yellowish brown, with dense small black spots; dorsal surface of body yellowish red or yellowish brown; belly and ventral surfaces of head and tail pinkish yellow or yellow, with more or less small black spots; iris reddish yellow or yellow.

Description of the holotype. Adult female, SVL 488 mm, TL 146 mm, TL/SVL 0.30, TL/total length 0.23; body elongated, laterally compressed; head distinct from neck; snout wide and blunt, projecting beyond lower jaw; rostral approximately as wide as high, slightly visible from above; nasal undivided; internasal elongated; prefrontal approximately trapezoidal, bordering orbits; frontal shield-shaped, slightly longer than wide; parietals large, longer than wide, gradually narrower posteriorly, median suture approximately equal to length of frontal; single loreal, not bordering orbit; 1 supraocular, approximately triangular; 1 preocular; 1 postocular and 1 elongated crescent-shaped subocular; 2 anterior temporals, 3 posterior temporals; 7 supralabials on each side, separated from eyes; 7 infralabials on left side and 8 infralabials on right side, anterior-most in contact with its opposite between mental and anterior chin-shields, first 4 in contact with anterior chin-shield on left side and first 5 in contact with anterior chin-shield on right side; 3 chin-shields pairs, first pair and third pair triangle and large, second pair small and elongate, chin-shields interlaced, no mental groove under chin and throat; ventral scales 190; precloacal plate undivided; subcaudals 89, paired; dorsal scales in 15 rows throughout, 1 row of vertebral scales distinctly enlarged, scales not keeled at anterior body, 5 rows of mid-dorsal scales keeled at middle and posterior body; 5 maxillary teeth on left side and 4 maxillary teeth on right side.

Coloration of the holotype in life. Dorsal surface of head dark yellowish red, scattered with dense, small black spots; dorsal surface of body yellowish red; 2 wide black stripes on dorsal neck from occipital region; a black stripe from lower posterior orbit downwards and backwards to junction of last 2 supralabials and a black dot on last supralabial on each side of head; approximately 47 vertical black stripes on each side of body, most stripes on different sides connected to each other on vertebrals; some irregular black stripes on each side of tail; belly and ventral surfaces of head and tail pinkish yellow with many small black spots; iris reddish yellow, pupil black.

Coloration of the holotype in preservative. Yellowish red dorsal surfaces of head and body faded to pinkish brown; black stripes on sides of body and tail still distinct; pinkish-yellow belly and ventral surfaces of head and tail faded to pinkish white; iris changed to grayish black and pupil changed to white.

Variations. Morphometric and meristic data for the type series of the new species are provided in Table 2. The paratypes resemble the holotype, except that the subocular and the postocular are fused on one side of the head. The posterior temporals vary in number from two to four. The dorsal surfaces of the head and body vary from light yellowish red to yellowish brown, the belly and ventral surfaces of the head and tail vary from yellowish white to yellow, the number of vertical black stripes on each side of the body vary from 46 to 53, and the iris varies from reddish yellow to yellow in the paratypes. In addition, the small black spots on the belly are much fewer in one paratype.

Etymology. The specific epithet *guanyinshanensis* refers to Guanyinshan Provincial Nature Reserve, where the new species was found.

**Figure 5:**
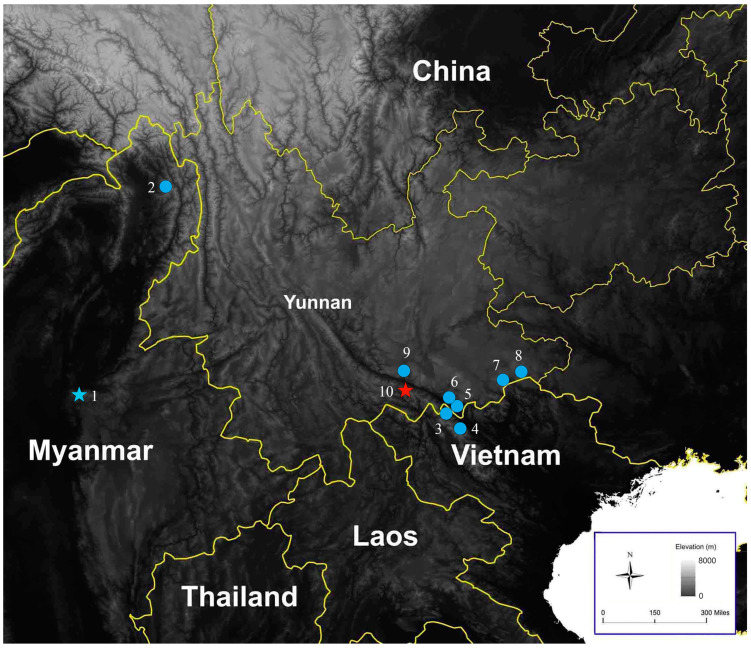
Map showing the type locality (shown by a blue star) of *Pareas hamptoni* sensu stricto, other confirmed distributions (blue dots) of *P. hamptoni* sensu stricto, and the type locality (indicated by a red star) of *Pareas guanyinshanensis* sp. nov. (**1**) Mogok, Mandalay, Myanmar; (**2**) Naung Mon, Putao, Kachin, Myanmar; (**3**) Bat Xat, Lao Cai, Vietnam; (**4**) Sa Pa, Lao Cai, Vietnam; (**5**,**6**) Hekou, Yunnan, China; (**7**,**8**) Malipo, Yunnan, China; (**9**) Jianshui, Yunnan, China; (**10**) Yuanyang, Yunnan, China.

Distribution. This new species is currently known only from Guanyinshan Provincial Nature Reserve in Yuanyang County, Honghe Prefecture, Yunnan Province, China (Figure 5).

**Figure 6:**
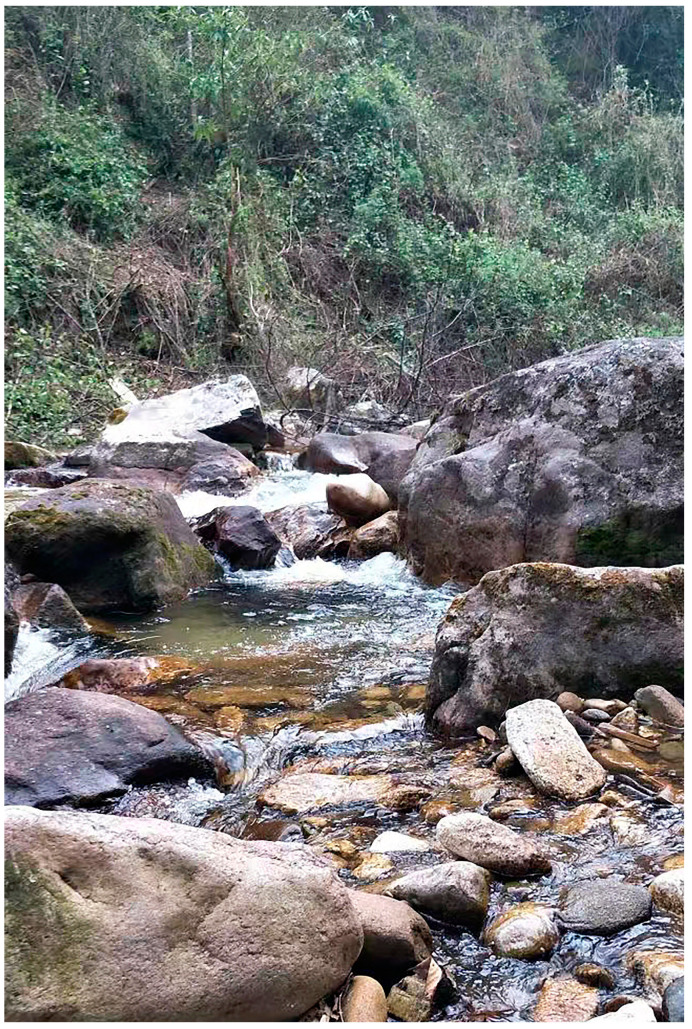
The habitat of *Pareas guanyinshanensis* sp. nov. at the type locality.

Habitat. All specimens of the new species were found on small branches or on the ground beside a stream at night, with forest and farmland nearby (Figure 6).

Comparison. *Pareas guanyinshanensis* sp. nov. can be distinguished from *P. abros* Poyarkov, Nguyen, Vogel & Orlov, 2022, *P. baiseensis* Wu, Gong, Huang & Xu, 2023, *P. berdmorei* Theobald, 1868, *P. carinatus* Wagler, 1830, *P. kuznetsovorum* Poyarkov, Yushchenko & Nguyen, 2022, *P. nuchalis* (Boulenger, 1900), and *P. temporalis* Le, Tran, Hoang & Stuart, 2021 by having one elongated crescent-shaped subocular or postocular and subocular that is fused into one semicircular scale (vs. 1–3 relatively short suboculars, with the subocular and postocular not fused).

*Pareas guanyinshanensis* sp. nov. can be distinguished from *P. andersonii* (Boulenger, 1888), *P. modestus* Theobald, 1868, *P. macularius* Theobald, 1868, and *P. margaritophorus* (Jan 1866) by its yellowish-red or yellowish-brown body background color (vs. body background colors of grey, dark grey, brownish gray, or completely black).

*Pareas guanyinshanensis* sp. nov. can be distinguished from *P. atayal* You, Poyarkov & Lin, 2015, *P. chinensis* (Barbour, 1912), *P. formosensis*, and *P. komaii* (Maki, 1931) by one row of vertebral scales being enlarged (vs. three rows that are enlarged).

*Pareas guanyinshanensis* sp. nov. can be distinguished from *P. boulengeri* (Angel, 1920), *P. stanleyi* (Boulenger, 1914), *P. vindumi* Vogel, 2015, and *P. xuelinensis* by the vertebral scales being enlarged (vs. not enlarged).

*Pareas guanyinshanensis* sp. nov. can be distinguished from *P. dulongjiangensis* Liu, Yang, Rao, Guo & Rao, 2023, *P. monticola* (Cantor, 1839), and *P. victorianus* Vogel, Nguyen & Poyarkov, 2021 by the loreal not being in contact with the orbit (vs. in contact with the orbit).

*Pareas guanyinshanensis* sp. nov. can be distinguished from *P. geminatus* by having more ventrals (189–192 vs. 170–188). *Pareas guanyinshanensis* sp. nov. can be distinguished from *P. iwasakii* (Maki, 1937) by having no distinct black stripe on the lateral head (vs. two clear thin black stripes on each side of the head).

*Pareas guanyinshanensis* sp. nov. can be distinguished from *P. kaduri* Bhosale, Phansalkar, Sawant, Gowande, Patel & Mirza, 2020, *P. niger* (Pope, 1928), *P. nigriceps* Guo & Deng, 2009, *P. tigerinus* Liu, Zhang, Poyarkov, Hou, Wu, Rao, Nguyen & Vogel, 2023, and *P. yunnanensis* (Vogt, 1922) by the dorsal surface of the head being yellowish red or yellowish brown with small black spots (vs. a large black blotch on the dorsal surface of the head or the dorsal surface of the head being solid black).

*Pareas guanyinshanensis* sp. nov. is phylogenetically closely related to *P. hamptoni* sensu stricto. However, *Pareas guanyinshanensis* sp. nov. can be distinguished from *P. hamptoni* sensu stricto by having fewer subcaudals (72–89 vs. 91–99) and a shorter tail (TL/SVL 0.26–0.30 vs. 0.32–0.37) (Table 6).

## 4. Discussion

Poyarkov et al. [5] divided the genus *Pareas* into two subgenera, namely, *Pareas* and *Eberhardtia*, and partitioned the subgenus *Eberhardtia* into four species groups, namely, the *P. chinensis*, *P. hamptoni*, *P. monticola*, and *P. margaritophorus* species groups. Currently, the *P. chinensis* species group consists of *P. baiseensis*, *P. boulengeri*, *P. chinensis*, and *P. stanleyi*; the *P. monticola* species group consists of only *P. monticola* and *P. victorianus*; and the *P. margaritophorus* species group consists of *P. andersonii*, *P. modestus*, *P. macularius*, and *P. margaritophorus*. The new species obviously belongs to the *P. hamptoni* species group. Therefore, the *P. hamptoni* species group should currently contain *P. atayal*, *P. dulongjiangensis*, *P. formosensis*, *P. geminatus*, *Pareas guanyinshanensis* sp. nov., *P. hamptoni*, *P. iwasakii*, *P. kaduri*, *P. komaii*, *P. niger*, *P. nigriceps*, *P. tigerinus*, *P. vindumi*, *P. xuelinensis*, and *P. yunnanensis*.

As the new species is phylogenetically the sister to and most resembles *Pareas hamptoni* sensu stricto, in order to compare the new species with *P. hamptoni* sensu stricto, we rely on the morphological data given by Ding et al. [11], combined with the original description [17] of this species and the new data in this paper, to obtain a relatively reliable morphological characterization of *P. hamptoni* sensu stricto. Although the morphological data of *P. hamptoni* sensu stricto given by Ding et al. [11] are slightly different from the original description [17] of this species, Ding et al. examined five specimens of *P. hamptoni* sensu stricto, which included the holotype of this species. Therefore, when the morphological data of *P. hamptoni* sensu stricto given by Ding et al. [11] are inconsistent with the original description [17] of this species, we adopt those given by Ding et al. [11].

*Pareas hamptoni* was once considered to be widely distributed, from Myanmar, Thailand, Laos, and Vietnam to southern China [18]. Wang et al. [6] restricted the distribution of *P. hamptoni* sensu stricto to Myanmar. Ding et al. [11] demonstrated that *P. hamptoni* sensu stricto is also distributed in northern Vietnam. Based on the specimens collected from Honghe and Wenshan prefectures in Yunnan Province, we confirmed the distribution of *P. hamptoni* sensu stricto in China (Figure 5) and describe a new species that is closely related to *P. hamptoni* sensu stricto. The distribution areas of these two species do not overlap, as the new species is from the southwest of the Red River, while all specimens of *P. hamptoni* sensu stricto in China were from the northeast of the Red River. But further downstream of the Red River, the situation is different. The record of *P. hamptoni* sensu stricto in Vietnam is from Lao Cai, which is located southwest of the Red River. In this way, *P. hamptoni* sensu stricto is distributed on both sides of the lower reaches of the Red River. However, in the relative upstream, *P. hamptoni* sensu stricto and the new species are distributed on different sides of the Red River, respectively.

## 5. Conclusions

**Figure 7:**
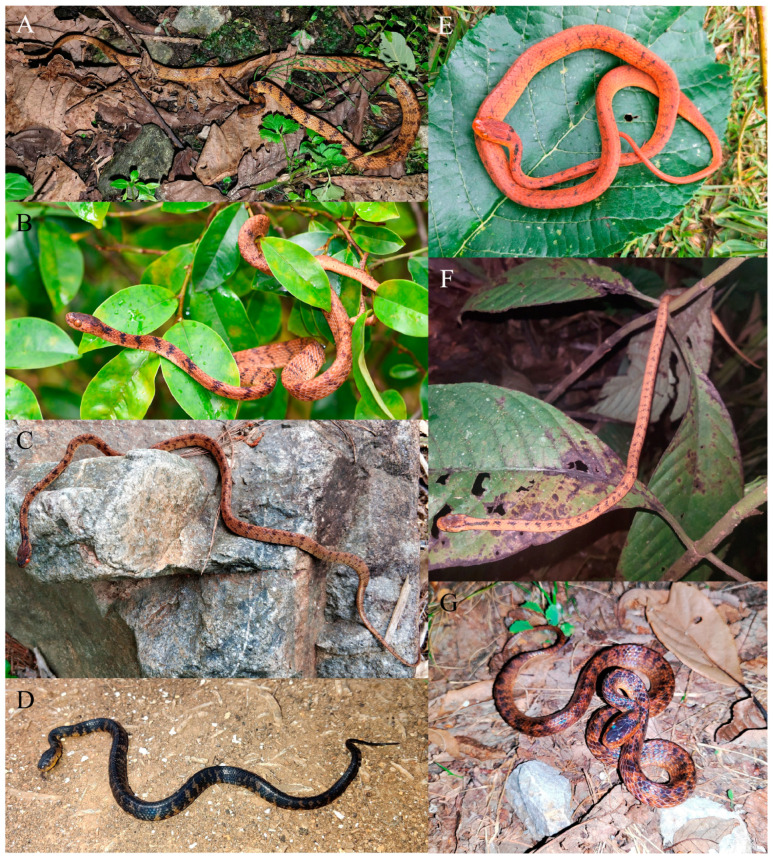
Species of the *Pareas hamptoni* complex in life. (**A**) *Pareas guanyinshanensis* sp. nov., from its type locality in Yuanyang County, Honghe Prefecture, Yunnan Province, China; (**B**) *P. hamptoni* sensu stricto, from Hekou County, Honghe Prefecture, Yunnan Province, China; (**C**) *P. formosensis*, from Nanping City, Fujian Province, China; (**D**) *P. niger*, from its type locality in Kunming City, Yunnan Province, China; (**E**) *P. xuelinensis*, from its type locality in Xuelin Township, Lancang County, Puer City, Yunnan Province, China; (**F**) *P. geminatus*, from its type locality in Jiangcheng County, Puer City, Yunnan Province, China; (**G**) *P. yunnanensis*, from its type locality in Dali City, Dali Prefecture, Yunnan Province, China.

A new *Pareas* species of the *P. hamptoni* complex (Figure 7) is described in this paper, based on three specimens collected from Guanyinshan Provincial Nature Reserve in Yuanyang County, Honghe Prefecture, Yunnan Province, China. Currently, the new species is known only from its type locality. The local ecological environment is relatively well maintained, and this species is less threatened at present.

A key to the members of the *Pareas hamptoni* complex:
1Dorsal head solid black........................................................................................................................2
Dorsal head yellowish brown to reddish brown.............................................................................32Dorsal body almost solid black........................................................................................*Pareas niger*
Dorsal body yellow to brownish red with black bars......................................*Pareas yunnanensis*3Vertebral scales not enlarged..................................................................................*Pareas xuelinensis*
Three rows of vertebral scales enlarged...............................................................*Pareas formosensis*
One row of vertebral scales enlarged................................................................................................44Subcaudals more than 91....................................................................*Pareas hamptoni* sensu stricto
Subcaudals less than 91.......................................................................................................................55Ventrals less than 188................................................................................................*Pareas geminatus*
Ventrals more than 189..................................................................*Pareas guanyinshanensis* sp. nov.

## Figures and Tables

**Table 1:** Samples used for molecular phylogenetic analysis in this study.

Species	Voucher	Locality	Cyt b	ND4
*Pareas guanyinshanensis* sp. nov.	KIZ 2023038	Yuanyang, Yunnan, China	PP215390	PP215399
*Pareas guanyinshanensis* sp. nov.	KIZ 2023039	Yuanyang, Yunnan, China	PP215389	PP215398
*Pareas guanyinshanensis* sp. nov.	KIZ 2023040	Yuanyang, Yunnan, China	PP215388	PP215397
*Pareas abros*	ZMMU R-16393	Song Thanh, Quang Nam, Vietnam	MZ712235	MZ712262
*Pareas andersonii*	CAS 235359	Natmataung, Chin, Myanmar	MT968772	MW287040
*Pareas atayal*	NMNS 05594	Beiheng, Taoyuan, Taiwan	KJ642124	MW287041
*Pareas baiseensis*	ANU 000220008	Baise, Guangxi, China	OQ054328	OQ054329
*Pareas berdmorei*	AUP 01573	Chiang Mai, Thailand	MZ712218	MZ712244
*Pareas boulengeri*	GP2923	Jiangkou, Guizhou, China	MK135090	MK805355
*Pareas carinatus*	KIZ 011972	Malaysia	MK135111	MK805376
*Pareas chinensis*	CIB 010140	Tianquan, Sichuan, China	JF827691	JF827669
*Pareas dulongjiangensis*	KIZ R201607	Gongshan, Yunnan, China	OQ718498	—
*Pareas formosensis*	NMNS 05637	Nantou, Taiwan	MW287060	MW287042
*Pareas formosensis*	H26-HAM01	Guangdong, China	MW287061	MW287043
*Pareas formosensis*	YBU 12015	Hainan, China	MK135068	MK805333
*Pareas formosensis*	GP4581	Jingning, Zhejiang, China	MK135072	MK805337
*Pareas formosensis*	YBU 12115	Rongjiang, Guizhou, China	MK135075	MK805340
*Pareas formosensis*	YBU 14508	Guangxi, China	MK135076	MK805341
*Pareas formosensis*	GP3696	Yanshan, Jiangxi, China	MH046857	MK805382
*Pareas formosensis*	ZMMU R-16333	Kon Chu Rang, Gia Lai, Vietnam	MW287066	MW287048
*Pareas formosensis*	ZMMU NAP-08868	Song Thanh, Quang Nam, Vietnam	MW287063	MW287045
*Pareas formosensis*	ZMMU R-16684	Phia Oac, Cao Bang, Vietnam	MW287062	MW287044
*Pareas formosensis*	ZMMU R-13709	Bidoup–Nui Ba, Lam Dong, Vietnam	MW287064	MW287046
*Pareas formosensis*	ZMMU R-14072	Chu Yang Sin, Dak Lak, Vietnam	MW287065	MW287047
*Pareas formosensis*	FMNH 255567	Pu Mat, Nghe An, Vietnam	AY425806	—
*Pareas geminatus*	DL2019072910	Jiangcheng, Yunnan, China	MW287067	—
*Pareas geminatus*	DL2019093001	Jiangcheng, Yunnan, China	MW287071	—
*Pareas geminatus*	DL2019093002	Jiangcheng, Yunnan, China	MW287072	—
*Pareas geminatus*	CIB 118021	Jiangcheng, Yunnan, China	MW287068	—
*Pareas geminatus*	CIB 118022	Jiangcheng, Yunnan, China	MW287069	—
*Pareas geminatus*	CIB 118023	Jiangcheng, Yunnan, China	MW287070	—
*Pareas geminatus*	KIZ L2020020	Jiangcheng, Yunnan, China	MW436707	—
*Pareas geminatus*	KIZ L2020024	Jiangcheng, Yunnan, China	MW436708	—
*Pareas hamptoni* sensu stricto	YPX 18604	Kachin, Myanmar	MK135078	MK805343
*Pareas hamptoni* sensu stricto	YPX 18219	Kachin, Myanmar	MK135077	MK805342
*Pareas hamptoni* sensu stricto	CAS 221489	Putao, Kachin, Myanmar	MW287077	—
*Pareas hamptoni* sensu stricto	ZMMU NAP-09087	Bat Xat, Lao Cai, Vietnam	MW287078	MW287054
*Pareas hamptoni* sensu stricto	ZMMU NAP-09088	Bat Xat, Lao Cai, Vietnam	MW287079	MW287053
*Pareas hamptoni* sensu stricto	ROM 38104	Sa Pa, Lao Cai, Vietnam	KX694896	—
*Pareas hamptoni* sensu stricto	KIZ 20210517	Malipo, Yunnan, China	PP215386	PP215395
*Pareas hamptoni* sensu stricto	KIZ L2020018	Malipo, Yunnan, China	PP215382	PP215391
*Pareas hamptoni* sensu stricto	KIZ 201903001	Hekou, Yunnan, China	PP215383	PP215392
*Pareas hamptoni* sensu stricto	KIZ 20210518	Hekou, Yunnan, China	PP215385	PP215394
*Pareas hamptoni* sensu stricto	KIZ 20210519	Hekou, Yunnan, China	PP215384	PP215393
*Pareas hamptoni* sensu stricto	KIZ 2023046	Jianshui, Yunnan, China	PP215387	PP215396
*Pareas iwasakii*	NMNS 05655	Ishigaki, Okinawa, Japan	KJ642160	—
*Pareas kaduri*	BNHS 3574	Lohit, Arunachal Pradesh, India	MW026190	—
*Pareas komaii*	NMNS 05618	Lijia, Taitung, Taiwan	KJ642185	MW287056
*Pareas kuznetsovorum*	ZMMU R-16802	Song Hinh, Phu Yen, Vietnam	MZ712232	MZ712258
*Pareas macularius*	ZMMU R-16629	Ban Mauk, Sagaing, Myanmar	MT968771	MW287057
*Pareas margaritophorus*	ZMMU NAP-09759	Suan Phueng, Ratchaburi, Thailand	MZ712217	MZ712243
*Pareas modestus*	MZMU 1293	Aizawl, Mizoram, India	MT968773	—
*Pareas monticola*	ZMMU R-16631	Ban Mauk, Sagaing, Myanmar	MW438296	MW438301
*Pareas niger*	KIZ 059339	Kunming, Yunnan, China	MW436706	—
*Pareas niger*	GP1294	Mengzi, Yunnan, China	MK135079	MK805344
*Pareas niger*	YBU 14251	Mengzi, Yunnan, China	MK135080	MK805345
*Pareas niger*	YBU 14252	Mengzi, Yunnan, China	MK135081	MK805346
*Pareas niger*	YBU 14253	Mengzi, Yunnan, China	MK135082	MK805347
*Pareas niger*	YBU 14288	Mengzi, Yunnan, China	MK135083	MK805348
*Pareas niger*	YBU 15100	Kaiyuan, Yunnan, China	MK135084	MK805349
*Pareas niger*	YBU 15114	Kaiyuan, Yunnan, China	MK135085	MK805350
*Pareas nigriceps*	CHS656	Tengchong, Yunnan, China	MK201455	—
*Pareas nuchalis*	FK 2626	Belait, Brunei Darussalam, Brunei	MZ603794	U49311
*Pareas stanleyi*	HM 2007-S001	Guilin, Guangxi, China	JN230704	JN230705
*Pareas temporalis*	ZMMU R-13656	Cat Loc, Lam Dong, Vietnam	MZ712238	MZ712265
*Pareas tigerinus*	KIZ 20210703	Menghai, Yunnan, China	OP752143	—
*Pareas victorianus*	CAS 235254	Natmataung, Chin, Myanmar	MW438300	MW438302
*Pareas vindumi*	CAS 248147	Lukpwi, Kachin, Myanmar	MW287080	MW287059
*Pareas xuelinensis*	KIZ XL1	Lancang, Yunnan, China	MW436709	—
*Pareas xuelinensis*	KIZ XL2	Lancang, Yunnan, China	MW436710	—
*Pareas yunnanensis*	KIZ 2022033	Dali, Yunnan, China	OP752146	—
*Pareas yunnanensis*	KIZ 2022034	Dali, Yunnan, China	OP752147	—
*Pareas yunnanensis*	KIZ 2022035	Dali, Yunnan, China	OP752148	—
*Pareas yunnanensis*	KIZ 2022036	Dali, Yunnan, China	OP752149	—
*Aplopeltura boa*	LSUHC 7248	Sepilok, Sabah, Malaysia	KC916746	U49312

**Table 2:** Measurements (in mm) and the scalation data of the type series of *Pareas guanyinshanensis* sp. nov. For abbreviations, see the Materials and Methods section.

	KIZ 2023038 Holotype ♀	KIZ 2023039 Paratype ♀	KIZ 2023040 Paratype ♀
SVL	488	482	540
TL	146	126	152
PrFBO	Yes	Yes	Yes
PreO	1/1	1/1	1/1
PosO	1/1	Fused/1	1/Fused
SubO	1/1	Fused/1	1/Fused
SPOF	No/No	Yes/No	No/Yes
ATem	2/2	2/2	2/2
PTem	3/3	4/4	2/2
SupL	7/7	8/8	7/7
InfL	7/8	7/6	7/7
LoBO	No	No	No
Vs	190	192	189
Prec	Undivided	Undivided	Undivided
Sc	89	72	80
DS	15-15-15	15-15-15	15-15-15
NED	1	1	1
NKD	0-5-5	0-5-7	0-5-5
Max	5/4	5/5	5/5

**Table 3:** Measurements (in mm) and scalation data of the specimens of *Pareas hamptoni* sensu stricto from China. For abbreviations, see the Materials and Methods section.

	KIZ 20210517 ♂	KIZ L2020018 Juvenile	KIZ 201903001 ♀	KIZ 20210518 Juvenile	KIZ 20210519 ♂	KIZ 2023046 ♂
SVL	458	276	360	306	459	532
TL	157	91	114	97	151	Incomplete
PrFBO	Yes	Yes	Yes	Yes	Yes	Yes
PreO	1/1	1/1	1/1	1/1	1/1	1/1
PosO	Fused/Fused	Fused/Fused	Fused/Fused	1/1	1/1	1/1
SubO	Fused/Fused	Fused/Fused	Fused/Fused	1/1	1/1	1/1
SPOF	Yes/yes	Yes/yes	Yes/yes	No/No	No/No	No/No
ATem	2/2	2/2	1/1	2/2	3/3	2/2
PTem	3/2	3/3	3/2	2/3	3/3	3/3
SupL	7/8	7/7	7/7	7/7	8/7	7/7
InfL	6/6	7/7	7/7	7/7	8/8	7/7
LoBO	No	No	No	No	No	No
Vs	188	188	187	193	191	186
Prec	Undivided	Undivided	Undivided	Undivided	Undivided	Undivided
Sc	92	97	94	95	91	Incomplete
DS	15-15-15	15-15-15	15-15-15	15-15-15	15-15-15	15-15-15
NED	1	1	1	1	1	1
NKD	0-3-5	0-3-3	0-3-5	0-3-3	0-3-5	0-5-7
Max	4/5	5/5	5/5	4/4	5/5	4/5

**Table 4:** Uncorrected p-distances (%), as calculated from Cyt b gene sequences. (1) *Pareas guanyinshanensis* sp. nov., (2) *P. abros*, (3) *P. andersonii*, (4) *P. atayal*, (5) *P. baiseensis*, (6) *P. berdmorei*, (7) *P. boulengeri*, (8) *P. carinatus*, (9) *P. chinensis*, (10) *P. dulongjiangensis*, (11) *P. formosensis*, (12) *P. geminatus*, (13) *P. hamptoni* sensu stricto, (14) *P. iwasakii*, (15) *P. kaduri*, (16) *P. komaii*, (17) *P. kuznetsovorum*, (18) *P. macularius*, (19) *P. margaritophorus*, (20) *P. modestus*, (21) *P. monticola*, (22) *P. niger*, (23) *P. nigriceps*, (24) *P. nuchalis*, (25) *P. stanleyi*, (26) *P. temporalis*, (27) *P. tigerinus*, (28) *P. victorianus*, (29) *P. vindumi*, (30) *P. xuelinensis*, and (31) *P. yunnanensis*.

	(1)	(2)	(3)	(4)	(5)	(6)	(7)	(8)	(9)	(10)	(11)	(12)	(13)	(14)	(15)	(16)	(17)	(18)	(19)	(20)	(21)	(22)	(23)	(24)	(25)	(26)	(27)	(28)	(29)	(30)
(1)																														
(2)	23.5																													
(3)	21.5	23.5																												
(4)	13.6	22.8	20.3																											
(5)	19.2	23.7	20.6	20.6																										
(6)	23.1	21.0	23.8	23.5	22.5																									
(7)	17.2	23.2	19.7	18.4	14.2	23.6																								
(8)	23.1	21.8	22.9	23.2	22.4	13.9	22.6																							
(9)	18.0	23.8	19.2	18.8	14.6	24.9	9.0	22.8																						
(10)	12.8	24.0	19.6	13.9	19.6	24.2	18.0	23.7	17.8																					
(11)	8.0	23.9	21.6	15.0	19.2	24.3	17.1	23.9	17.8	13.4																				
(12)	7.5	22.9	22.2	14.1	19.0	22.9	17.4	23.2	19.0	12.8	9.5																			
(13)	4.2	23.5	21.7	14.0	18.9	23.3	17.2	23.5	18.3	12.6	8.0	7.4																		
(14)	13.1	23.4	20.5	7.0	19.3	24.4	17.4	24.3	18.3	14.0	14.5	14.5	13.7																	
(15)	12.3	24.5	20.2	14.7	19.8	24.5	19.4	22.2	18.8	9.1	13.4	13.4	12.7	14.8																
(16)	14.3	23.2	19.5	8.6	19.5	23.9	18.0	23.9	18.2	13.7	14.7	14.9	14.4	8.1	15.5															
(17)	22.7	21.2	23.8	23.2	23.3	13.2	22.6	13.2	23.0	24.2	23.3	23.1	23.2	24.4	22.9	24.3														
(18)	18.7	23.9	14.5	18.9	18.5	22.7	18.4	21.7	17.6	18.3	19.8	20.3	18.9	19.5	19.6	19.2	22.6													
(19)	19.9	24.7	16.2	18.9	20.0	24.2	19.2	22.8	18.7	17.9	19.6	20.5	19.4	19.0	20.1	18.4	23.1	14.9												
(20)	20.4	23.5	12.0	18.4	19.1	24.2	19.2	24.1	18.8	18.6	20.3	20.1	19.7	19.8	19.2	17.9	24.4	12.0	14.8											
(21)	18.6	22.7	18.7	17.7	20.3	21.7	18.3	22.9	18.5	17.6	19.5	19.5	18.6	17.7	18.7	18.1	21.9	17.1	18.9	18.7										
(22)	5.7	22.6	20.6	14.1	19.0	23.5	17.6	23.3	17.9	12.2	8.3	6.9	6.0	13.6	12.1	14.6	22.7	18.6	19.9	19.0	18.9									
(23)	12.4	23.6	18.8	15.7	16.9	22.9	16.9	22.9	16.2	10.3	13.3	13.5	12.6	16.1	9.7	16.2	23.9	19.3	17.8	16.4	18.8	12.6								
(24)	25.0	21.1	24.3	23.4	23.4	21.2	24.3	21.2	24.1	24.6	24.6	25.1	24.8	24.3	25.0	23.5	20.4	23.3	24.6	24.5	22.5	25.2	23.8							
(25)	19.2	25.7	20.4	19.2	16.3	25.2	15.7	24.7	15.4	19.2	19.5	19.5	18.7	18.9	20.3	17.3	24.9	18.6	19.5	19.4	18.8	19.6	19.0	24.0						
(26)	23.8	13.3	23.8	23.6	23.3	20.6	22.5	20.1	21.9	25.1	24.6	23.9	23.7	23.6	24.8	24.2	20.1	23.4	24.8	23.6	23.0	23.6	24.1	20.2	24.1					
(27)	12.1	23.0	19.3	14.3	19.7	24.1	19.0	23.3	18.7	10.3	12.9	12.4	11.8	14.2	10.7	14.0	24.3	18.6	19.5	18.2	18.7	11.4	4.3	25.0	19.3	24.8				
(28)	19.1	24.3	20.6	19.5	19.2	22.8	19.1	22.9	17.5	18.0	18.1	18.6	18.6	19.9	18.4	19.3	22.9	19.0	20.6	19.3	14.3	17.8	19.1	24.7	19.0	24.5	17.9			
(29)	11.0	24.4	20.8	14.7	19.5	24.4	18.4	24.2	17.7	12.6	12.5	12.4	11.5	14.5	12.3	15.0	23.8	19.0	19.9	19.9	17.9	11.0	12.3	24.7	19.4	25.3	11.9	17.8		
(30)	8.3	23.1	21.3	13.8	20.5	24.7	16.9	24.3	18.7	13.3	8.9	6.0	8.3	13.9	13.2	14.8	24.6	19.7	20.0	20.2	19.6	7.3	12.5	25.9	19.5	24.7	12.3	18.8	12.6	
(31)	7.1	23.2	22.1	14.4	19.1	24.1	16.7	23.5	18.2	12.9	8.6	4.1	6.2	13.9	12.4	14.7	23.7	20.1	20.1	20.6	19.6	6.4	12.8	24.9	19.5	23.9	11.7	18.7	11.5	6.2

**Table 5:** Uncorrected p-distances (%), as calculated from the ND4 gene sequences. (1) *Pareas guanyinshanensis* sp. nov., (2) *Pareas abros*, (3) *P. andersonii*, (4) *P. atayal*, (5) *P. baiseensis*, (6) *P. berdmorei*, (7) *P. boulengeri*, (8) *P. carinatus*, (9) *P. chinensis*, (10) *P. formosensis*, (11) *P. hamptoni* sensu stricto, (12) *P. komaii*, (13) *P. kuznetsovorum*, (14) *P. macularius*, (15) *P. margaritophorus*, (16) *P. monticola*, (17) *P. niger*, (18) *P. nuchalis*, (19) *P. stanleyi*, (20) *P. temporalis*, (21) *P. victorianus*, and (22) *P. vindumi*.

	(1)	(2)	(3)	(4)	(5)	(6)	(7)	(8)	(9)	(10)	(11)	(12)	(13)	(14)	(15)	(16)	(17)	(18)	(19)	(20)	(21)
(1)																					
(2)	18.6																				
(3)	19.0	20.4																			
(4)	16.7	22.3	19.1																		
(5)	18.1	21.8	18.0	20.2																	
(6)	21.0	19.5	21.5	22.0	25.5																
(7)	18.7	21.1	17.8	18.4	12.1	22.8															
(8)	21.5	18.9	23.0	21.1	22.4	14.3	21.0														
(9)	18.3	21.1	18.3	20.1	12.2	22.8	10.1	22.6													
(10)	9.1	18.8	19.6	16.2	17.1	20.7	19.3	21.3	17.5												
(11)	5.0	19.4	19.0	16.4	17.5	21.2	17.9	20.9	16.5	8.7											
(12)	17.8	21.9	19.7	7.5	20.8	22.3	19.0	21.6	19.2	16.2	17.5										
(13)	19.1	19.9	21.3	21.1	22.8	14.1	19.8	15.0	21.7	19.7	18.9	21.3									
(14)	20.3	22.5	11.8	19.8	18.6	22.9	19.5	23.2	20.2	20.9	20.4	20.8	23.7								
(15)	19.9	20.8	14.0	19.6	18.9	21.3	18.6	21.0	20.8	19.6	20.1	21.0	20.2	14.7							
(16)	18.7	21.7	19.6	18.9	19.3	21.6	18.2	20.7	18.4	20.0	18.5	20.5	19.5	22.0	21.4						
(17)	9.5	19.8	18.8	16.0	18.0	22.2	17.6	21.4	17.1	8.8	9.0	16.2	21.3	20.4	18.6	19.7					
(18)	21.3	17.4	19.9	23.4	22.8	18.2	22.3	18.8	22.5	20.3	21.1	23.7	19.5	23.4	21.1	21.3	21.4				
(19)	20.3	21.6	18.0	19.5	16.2	23.9	14.8	22.5	16.6	19.3	20.4	18.6	22.4	18.0	19.5	19.2	19.1	23.0			
(20)	19.0	9.4	20.0	19.9	20.8	19.3	20.2	19.2	18.7	18.9	19.4	19.6	18.9	21.9	19.6	19.5	18.0	17.4	20.1		
(21)	17.6	20.4	18.5	18.4	18.4	22.9	18.3	20.8	18.6	18.0	17.4	19.0	21.7	20.5	20.2	12.8	18.6	23.5	19.5	18.3	
(22)	12.5	19.0	16.3	13.9	14.8	21.1	15.6	20.4	15.7	12.8	13.0	14.2	19.2	18.0	18.6	17.4	13.6	20.1	16.5	18.5	15.6

**Table 6:** Comparison between *Pareas guanyinshanensis* sp. nov. and *P. hamptoni* sensu stricto. Measurements are in mm. The data for *P. hamptoni* sensu stricto were obtained by combining those of the original description, Ding et al. [11], and this study.

	*Pareas guanyinshanensis* sp. nov.	*Pareas hamptoni* Sensu Stricto
SVL (adult)	482–540	360–532
TL (adult)	126–152	114–157
TL/SVL	0.26–0.30	0.32–0.37
PrFBO	Yes	Yes
PreO	1	1
PosO	Fused or 1	Fused or 1
SubO	Fused or 1	Fused or 1
SPOF	Yes or No	Yes or No
ATem	2	1–3
PTem	2–4	2–3
SupL	7–8	7–8
InfL	6–8	6–9
LoBO	No	No
Vs	189–192	185–195
Prec	Undivided	Undivided
Sc	72–89	91–99
DS	15-15-15	15-15-15
NED	1	1
NKD	0–5	0–9
Max	4–5	4–5

## Data Availability

All data are presented in this article.
